# Colonic Basidiobolomycosis—An Unusual Presentation of Eosinophilic Intestinal Inflammation

**DOI:** 10.3389/fped.2020.00142

**Published:** 2020-04-21

**Authors:** Elena Kurteva, Alasdair Bamford, Kate Cross, Tom Watson, Catherine Owens, Fanlek Cheng, John Hartley, Kathryn Harris, Elizabeth M. Johnson, Keith Lindley, Samantha Levine, Jutta Köglmeier

**Affiliations:** Great Ormond Street Hospital for Children, London, United Kingdom

**Keywords:** basidiobolomycosis, colon, eosinophilic inflammation, intestine, children

## Abstract

Basidiobolomycosis is a rare fungal disease caused by *Basidiobolus ranarum*. Involvement of the gastrointestinal tract is unusual and poses both a diagnostic and therapeutic challenge, as clinical signs are non-specific and predisposing risk factors are lacking. It can mimick inflammatory bowel disease, primary immunodeficiency, or a malignancy and should be considered in patients who do not respond to standard therapy. We present the case of a 22 months old boy with confirmed colonic Basidiobolomycosis, who presented with severe eosinophilic inflammation of the gastrointestinal tract. Panfungal PCR performed on DNA extracted directly from a tissue sample confirmed the presence of *Basidiobolus*. He made a full recovery with a combination of surgery and prolonged targeted antifungal medication.

## Introduction

Eosinophilic infiltration of the gastrointestinal tract is associated with parasitic infections, vasculitis, connective tissue disorders, drug hypersensitivity reactions, and inflammatory bowel disease.

*Basidiobolus ranarum* is a known cause of subcutaneous entomophthoromycosis (previously loosely classified as zygomycosis) and has been recognized to cause gastrointestinal infections and gastrointestinal eosinophilia in rare cases.

The colon is the most commonly involved part of the gastrointestinal system and affected individuals are usually immunocompetent. Diagnosis is dependent on imaging, histopathology, culture, and more recently molecular detection methods. Treatment requires prolonged antifungal therapy, together with surgical resection wherever possible.

## Case Presentation

A 20 months old boy originating from the United Arabic Emirates presented to his local pediatric department with a 2 weeks history of abdominal pain, vomiting, and streaks of fresh blood in the stool. Prior to the onset of his symptoms he had been fit and well apart from an episode consistent with hand foot and mouth disease in the preceding 4 weeks, which settled with conservative management. He was the first child of unrelated parents. His mother suffered from type 1 diabetes but there was otherwise no family history of note. Initial blood tests demonstrated an elevated IgE (906 kU/L) and leukocytosis (22.1 × 10^9^/L) with raised eosinophil count (19.3 × 10^9^/L). Renal, liver function, albumin, CRP, and ESR were normal. An ultrasound of the abdomen was unremarkable. He had lost 2 kg in weight but was previously thriving well along the 75th centile on an age appropriate diet.

His height was in between the 75th and 91st centile. He was commenced on Omeprazole and received a 10 days course of oral Metronidazole which resulted in resolution of vomiting and improved weight gain, but abdominal pain and blood in the stool continued.

After discontinuation of antibiotic therapy he started to vomit profusely requiring intravenous fluids.

**Figure 1:**
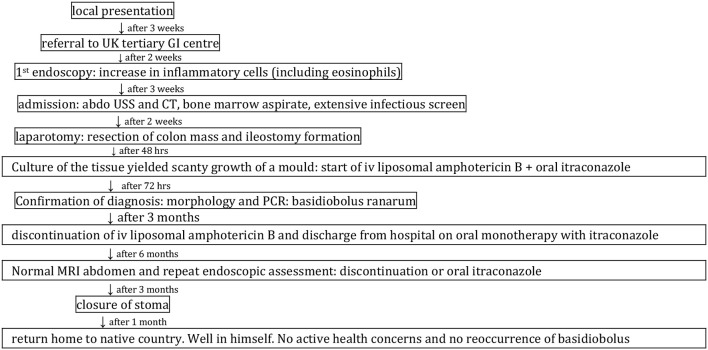
Timeline.

The child was referred to Great Ormond Street Hospital 2 weeks later for upper and lower gastrointestinal endoscopy. The timeline from presentation to completion of his treatment is outlined in Figure 1. During this period he had developed worsening abdominal pain particularly after eating and his oral intake had reduced significantly. He continued to pass fresh blood in the stool and he had lost 2 kg in weight again.

He had a mildly elevated ESR of 20 mm/h (normal <10), thromobocytosis of 509 × 10^9^/L (150–450) and a significantly elevated white cell count 27.23 × 10^9^/L) with a prominent eosinophophilia (6.79 × 10^9^) and high IgE (1,574 kU/L) with specific IgE positive to soya, wheat, and cow's milk.

His fecal calprotectin was markedly elevated to 2,420 mg/Kg (<50). The initial histological assessment of endoscopic biopsies showed a chronic gastritis with up to 15 eosinophils per high power field (HPF), normal duodenal and ileal biopsies and mild patchy architectural distortion with some mucosal oedema and increase in lamina propria inflammatory cells including eosinophils in the colon. Potential differential diagnoses considered at the time were a post infectious process or inflammatory bowel disease. In view of the positive specific IgE he was commenced on an exclusion diet of cow's milk, soya and wheat and started on oral Prednisolone (2 mg/kg daily), sodium cromoglycate (10 mg/kg four times daily), and sulphasalazine (10 mg/kg four times daily).

After an initial improvement he was subsequently admitted urgently with an acute generalized deterioration, high fever, lethargy, and severe abdominal pain. He was commenced on iv methylprednisolone (2 mg/kg) in view of his previous endoscopic findings of a panenteric eosinophilic infiltrate of the gut and poor response to oral medication and dietary manipulation. Intravenous amikacin and piperacillin/tyazobactam were also started as per local protocol for suspected sepsis.

**Figure 2:**
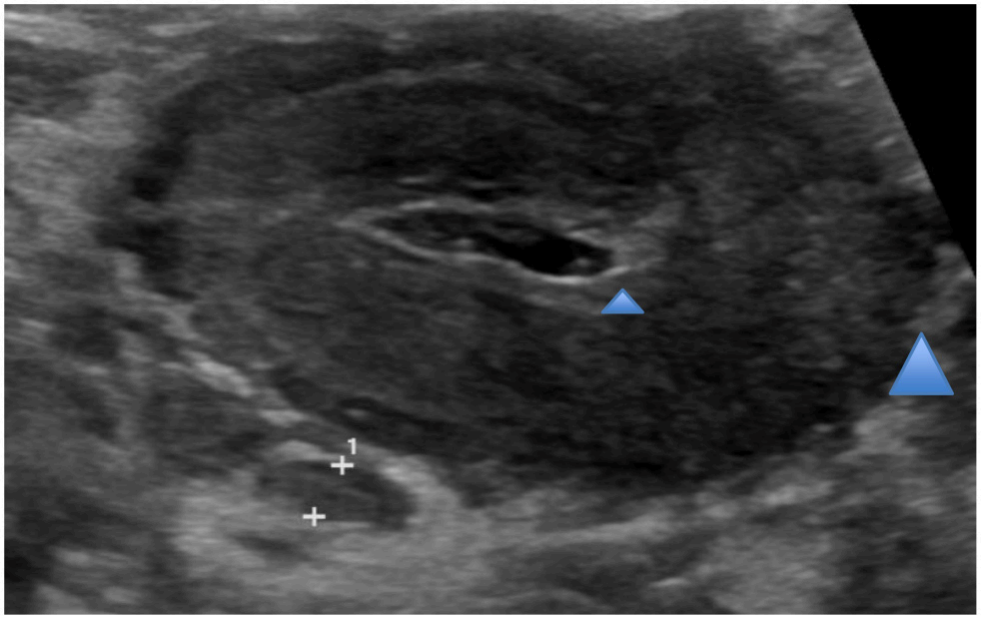
Abdominal ultrasound. Marked thickening of colon loops within mid epigastric and left upper quadrant representing transverse (see blue arrows) and descending colon.

He continued to have high temperatures up to 40.5°C on a daily basis with a persistent rise of CRP and ESR, thrombocytosis and significant elevation of the total white cell count, eosinophils and neutrophils. A repeat ultrasound of the abdomen showed a markedly thickened transverse and descending colon (see Figure 2). He became increasingly unwell with more abdominal distension and a palpable mass on the left side of his abdomen.

**Figure 3:**
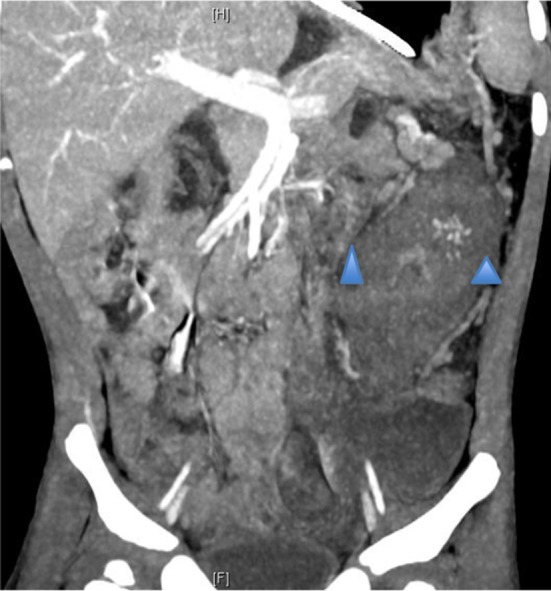
Abdominal CT with contrast. Marked bowel wall thickening of left colon (see blue arrows) extending from the distal transverse to the descending colon. There is an area of spared bowel in the mid descending colon. The thickened bowel is homogenous in attenuation, with relative hyper enhancement of the mucosa. There is abrupt transition between normal and abnormal bowel (shouldering). There is involvement of the serosa with multiple serosal nodules and infiltration of the pericolonic fat.

**Figure 4:**
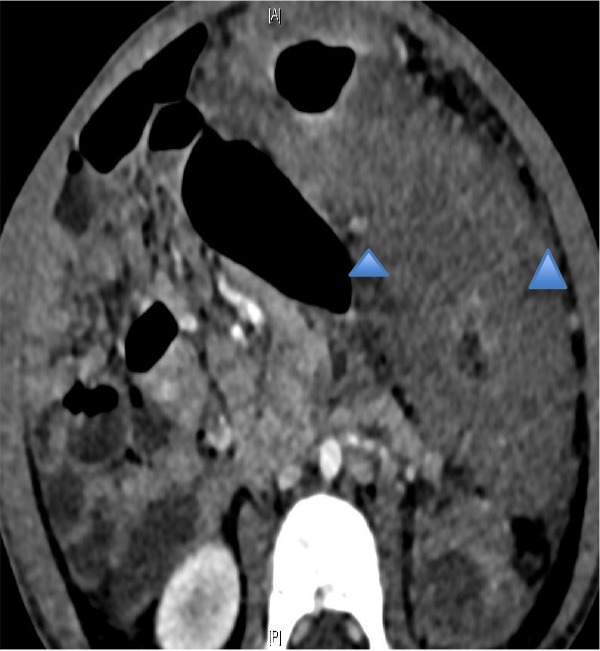
Same as Figure 3.

A subsequent urgent CT abdomen with contrast confirmed a markedly abnormal transverse and left colon with extensive low attenuation bowel wall thickening (see Figures 3, 4). A malignant process such as a lymphoma, an infectious colitis or vasculitis were considered.

His immunosuppression was stopped and he underwent an ultrasound guided interventional radiology bowel biopsy, bone marrow aspirate with trephine and Hickman catheter insertion.

He was started on parenteral nutrition and gut rest and albendazole was added empirically to cover the possibility of a parasitic infection.

An extensive infection screen was initiated:

**Blood:** Cultures, echinococcus, hydatid, schistosoma, strongyloides, toxocara, toxoplasma (IgM/IgG), quantiferon, PCR for enterovirusm paraechovirus, HSV, VZV, parvovirus, adenovirus, CMV, EBV, RIPL (rare and imported pathogens panel).
**Urine:** Microscopy and culture.
**Stool:** Microscopy, culture, ova, cysts, parasites, cryptosporidium, PCR for CMV, norovirus, rotavirus, adenovirus, astrovirus, sapovirus, and clostridium difficile.

Investigations were also sent to exclude a primary immunodeficiency and haemophagocytic lymphohistiocytosis (HLH).

**Figure 5:**
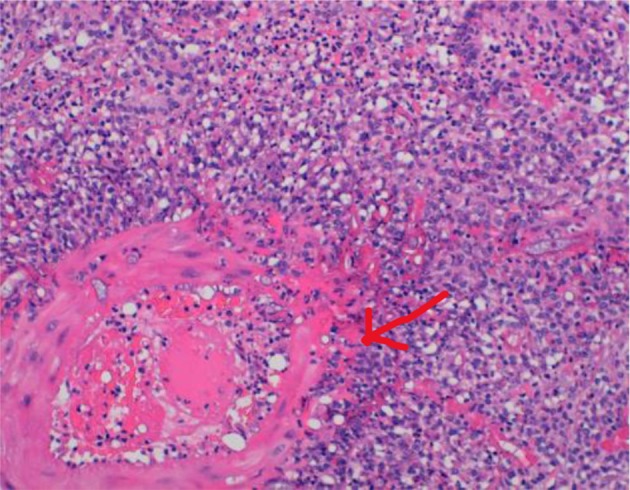
HE stain of bowel biopsy. Cores of fibrous tissue with extensive necrotizing granulomatous inflammation with many foreign body type multinucleated giant cells (see red arrow) and large numbers of eosinophils.

The bone marrow aspirate and trephine were rich in eosinophils. The histological examination of the bowel biopsies was highly suspicious of an infectious process with sheets of eosinophils, giants cells and granulomas with a necrotic center (see Figure 5). At the time of biopsy these samples were not sent for microbiological culture or molecular diagnosis by PCR, which may have allowed an earlier diagnosis, although unlikely as the site of the established infection is usually deeper than the superficial mucosa.

**Figure 6:**
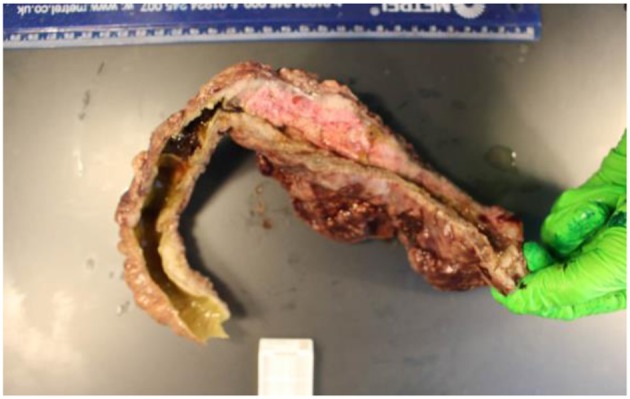
Resected left hemicolon.

His condition deteriorated further with worsening abdominal distension, evolving palpable colonic mass and ongoing pyrexia with further rise of the inflammatory markers. He was referred to the surgical team for a laparotomy. At the time of surgery a large heterogeneous mass was found arising from the left and transverse colon including the splenic flexure with omental wrapping and adherence to the sigmoid colon. The small bowel and proximal colon appeared macroscopically normal. A partial colectomy involving the distal transverse to sigmoid colon was performed and a colostomy formed. The resected specimen (see Figure 6) was sent for microbiology and histology and a peritoneal swab sent for microbiological analysis.

Culture of the tissue yielded scanty growth of a mold after 48 h incubation (on the MacConkey and Sabouraud plates), felt to be morphologically consistent with *Basidiobolus ranarum* at 72 h, although there are several morphologically similar species in the genus which all produce the characteristic ballistospores in culture; in-house panfungal PCR performed on DNA extracted directly from the tissue sample confirmed the presence of *Basidiobolus* sp. The isolate was referred to the Public Health England Mycology Reference Laboratory, Bristol, which undertook sensitivity testing by means of a reference broth microdilution method for molds CLS1 M38AL and confirmed the diagnosis. Panfungal PCR directly on the tissue was also positive for *Basidobolus ranarum*.

**Figure 7:**
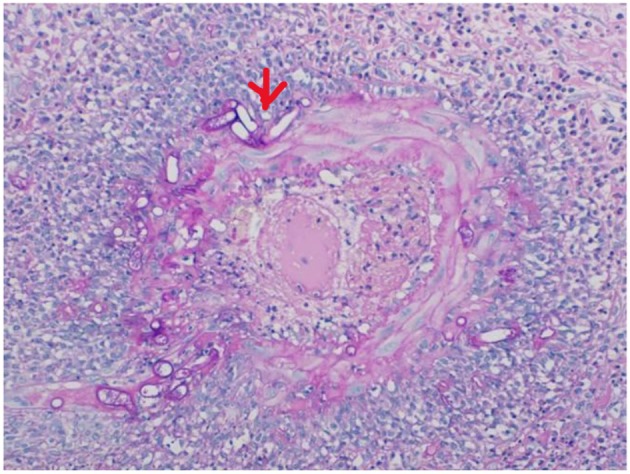
HE stain of the excised bowel. Splendore Hoeppli (see red arrow).

**Figure 8:**
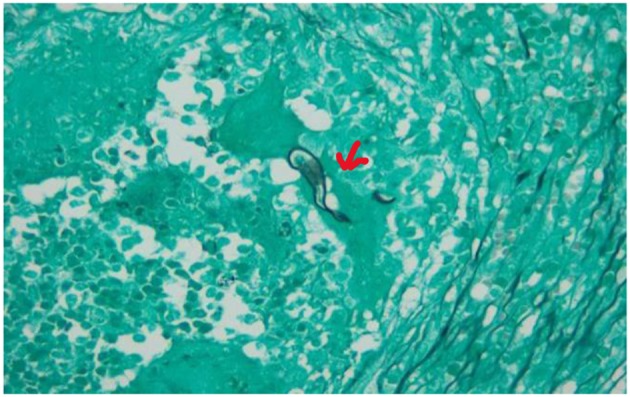
Grocott stain of the excised bowel. Basidiobolus hyphae (see red arrow). Typically thin-walled, septated hyphae, haphazardly branching, surrounded by eosinophilic material.

The histological examination of the excised bowel specimen showed a fungal infiltrate (see Figures 7, 8).

A diagnosis of colonic basidiobolomycosis was made.

Treatment with iv liposomal amphotericin B (3 mg/kg once daily) and voriconazole (9 mg/kg twice daily for 24 h loading dose, followed by 8 mg/kg twice daily maintenance) was commenced whilst awaiting confirmation of susceptibility test results. The child subsequently improved significantly and was weaned off parenteral nutrition back onto a normal diet. The isolate was found to be susceptible to amphotericin and itraconazole and his medication was hence modified accordingly (see Tables 1, 2).

**Table 1:** MICs (in mg/L) of isolate and derived clinical interpretation.

Echinocandins	MIC: 1.0	S
Isavuconazole	MIC: 0.25	S
Amphotericin B	MIC: 0.5	S
Itraconazole	MIC: 1.0	S
Fluconazole	MIC: > 32	R
Posaconazole	MIC: 8.0	R
Voriconazole	MIC: > 16	R

**Table 2:** Trough Itraconazole levels in mg/l.

	**OH-itraconazole**	**Itraconazole**
At 1 month	1.31	0.88
At 4 months	1.39	0.81
At 6 months	1.88	0.78

Intravenous lipososomal amphotericin B was continued and oral itraconazole commenced (initially 5 mg/kg twice daily, increased to 8 mg/kg twice daily to achieve and maintain target levels) aiming for combined (itraconazole and hydroxyl-itraconazole) blood levels of 2–3 mg/l.

He developed a palpable mass close to the stoma site shortly afterwards and ultrasound demonstrated a mass which MRI demonstrated to be an abscess. He had a prolonged course of antibiotics in addition to the antifungal therapy and the mass resolved.

Liposomal amphotericin B was discontinued after 3 months of intravenous therapy and itraconazole continued as oral monotherapy for a further 6 months. Toxicity from this regimen was minimal. Therapeutic trough itraconazole levels were achieved and maintained throughout the duration of therapy (see Table 2).

During this time the child started to pass mucous and blood per rectum and he complained about abdominal pain again with poor appetite and abnormal posturing. An ultrasound did not reveal pathology but upper and lower gastrointestinal endoscopy demonstrated a healing duodenal ulcer. The bowel behind the stoma looked healthy and there was only mild erythema around the surgical clips. The histological examination of the endoscopic biopsy specimen showed oesophagitis with chronic inflammation and up to 18 eosinophils per high power field (HPF), gastritis with eosinophils, many with degranulation and 28 eosinophils/HPF in the duodenum. He was commenced on lansoprazole and his symptoms resolved completely.

Following normal repeat endoscopic assessment and MRI of the abdomen antifungal therapy was discontinued after 6 months and the stoma reversed a further 3 months later.

He has remained well since with good weight gain and no reoccurrence of his symptoms. Throughout his treatment the child and his parents received psychological support. Particularly the parents experienced high levels of anxiety at the beginning of their son's treatment whilst no diagnosis was made. The boy regressed in his emotional development. He had started to potty train prior to the start of his symptoms but required nursing in nappies until his treatment was completed. Since the stoma has been closed he has made excellent progress. The family has returned to their home country and the boy has successfully integrated into nursery school.

## Discussion

Pediatric colonic basidiobolomycosis is a rare fungal infection of the colon caused by *Basidiobolus ranarum* (1). The case presented is unique as it is the first report of a child from the United Arab Emirates and also the first report of the use of pan fungal PCR followed by sequencing of the product to rapidly confirm the diagnosis not only by molecular confirmation of the isolate identification but also by detection of fungal DNA, directly from the colonic biopsy material.

It demonstrated the importance of including fungal infection in the differential diagnoses of eosinophilic inflammation of the gut, the utility of sending fresh specimens from bowel surgery for culture and highlights that histology of endoscopic surface mucosal biopsy is unlikely to be sufficient for diagnosing gastrointestinal basidiobolomycosis, as the associated mucosal eosinophilia is non-specific and seen in other disorders of the gastrointestinal tract such as inflammatory bowel disease.

*Basdidiobolus ranarum* is an environmental saprophytic fungus found worldwide in soil, decaying organic matter and the gastrointestinal tracts of amphibians, fish, reptiles and insectivorous bats (2). Infection in humans is endemic in tropical and subtropical regions of Africa, Asia, and Latin America. Subcutaneous mycoses caused by *Basidiobolus ranarum* has been known for a long time and infects predominantly the limbs of children and young adults. Although the subcutaneous form is characteristic, diagnosis is often delayed due to its slow and painless progression, poor response to antibiotic and anti-inflammatory medication and unfamiliarity of the skin lesions (3).

Colonic infection is extremely rare and presents both a diagnostic and therapeutic challenge. Affected patients usually present without any underlying risk factors such as immunodeficiency and all age groups are susceptible (4).

The condition may mimic inflammatory bowel disease (IBD) demonstrated by case presentations in the literature, where gastrointestinal basidiobolomycosis was mistaken for Crohn's disease (5, 6). Raised levels of Th2-type cytokines interleukin 4 and 10 and tumor necrosis factor alpha, which are present in patients with IBD have also been found in the serum of patients with gastrointestinal basidiobolus infection leading to further diagnostic confusion (4). Treatment failure to anti-inflammatory, immunosuppressive and immune modulatory therapy used in IBD should alert the physician to consider an alternative cause for the dense eosinophilic inflammation characteristic of fungal involvement of the colon.

By the late 1990s only a few cases of *Basidiobolus ranarum* infection of the gastrointestinal tract had been published (7). Since then a total of around 100 cases can be found in the medical literature of which 28 are children (8–10). All of the described pediatric patients were male. There is evolving evidence that gastrointestinal infections with *Basidiobolus ranarum* are increasingly recognized worldwide with 19 cases diagnosed in the United States (11).

Owing to its none-specific signs and symptoms, delay in diagnosis is frequent and often associated with an increased morbidity (12). Fatalities have been reported (13).

It is poorly understood how the fungus is introduced into the host's gastrointestinal tract but likely occurs due to ingestion of contaminated soil, animal feces, or food (8). Our patient was found eating soil in a public garden prior to the onset of his symptoms which may have been the route of infection. The use of contaminated toilet leaves for cleaning of the skin after defecation has also been considered as a possible route of entry for the subcutaneous and perineal presentation of infection as there is rarely overt evidence of trauma (14). The current experience in how to best treat patients with colonic basidiobolomycosis is limited to a small number of cases available in the literature. Surgical resection together with prolonged antifungal therapy appear to be the best available therapeutic option and can lead to complete resolution of the disease (8).

Case reports predominantly have used itraconazole and the organism is commonly found to be susceptible to this. Use of other antifungal agents including voriconazole, posaconazole, liposomal amphotericin, and potassium iodide has been reported. Resistance to amphotericin has been described as common and there have been poor outcomes associated with its use (in contrast with the case reported here, in which amphotericin had a low MIC for the organism).

Ketoconazole and fluconazole have also been found to be effective in some cased of subcutaneous basidiobolomycosis (15).

## Conclusion

Infection with basidiobolus ranarum in children is rare cause of severe eosinophilic inflammation of the gastrointestinal tract. It can mimick inflammatory bowel disease, primary immunodeficiency or a malignancy and should be considered in patients who do not respond to standard therapy. The outcome can be fatal as diagnosis and subsequent therapy is often delayed. There is a good prognosis with a combination of surgery and prolonged targeted antifungal medication.

Physicians, pathologists, and surgeons involved in the care of children should become familiar with this condition and a multidisciplinary approach is essential to achieve a favorable clinical course. Restoration of complete health can be achieved if treatment is initiated early.

## Data Availability Statement

The datasets generated for this study are available on request to the corresponding author.

## Ethics Statement

Ethical review and approval was not required for the study on human participants in accordance with the local legislation and institutional requirements. Written informed consent to participate in this study was provided by the participants' legal guardian/next of kin. Written informed consent was obtained from the minor(s)' legal guardian/next of kin for the publication of any potentially identifiable images or data included in this article.

## Author Contributions

EK, AB, KC, TW, CO, FC, JH, KH, EJ, KL, and SL have made substantial contributions to the data collection from medical records, the analysis and interpretation of data for the work. In addition AB supported JK in making the diagnosis and adjusting antifungal treatment and KC operated on the child. JK holds the intellectual property of the work and was the lead clinician of the patient. She supported EK in writing the first and final version of the manuscript, interpretation of data, literature review, advised on the design, and writing up the paper. The included in this case review was admitted to Great Ormond Street Hospital under JK and written consent to present the case study was taken on the ward by the parents who hold parenteral responsibility. JK took part at drafting the work and final approval of the version to be published, agrees to be accountable for all aspects of the work in ensuring that questions related to the accuracy or integrity of any part of the work are appropriately investigated and resolved.

## Conflict of Interest

The authors declare that the research was conducted in the absence of any commercial or financial relationships that could be construed as a potential conflict of interest.
